# Supplementary material for: The proteasome modulates endocytosis specifically in glomerular cells to promote kidney filtration
Source: Nat Commun. 2024 Mar 1;15:1897. doi: 10.1038/s41467-024-46273-0 (PMC10907641; doi:10.1038/s41467-024-46273-0)
Supplement: Supplementary file 1 — Supplementary Information [file 41467_2024_46273_MOESM1_ESM.pdf]

Supplementary Material

Titel *The Proteasome Modulates Endocytosis Specifically in Glomerular Cells to Promote Kidney Filtration*

Authors Wiebke Sachs, Lukas Blume, Desiree Loreth, Lisa Schebsdat, Favian Hatje, Sybille Koehler, Uta Wedekind, Marlies Sachs, Stephanie Zielinski, Johannes Brand, Christian Conze, Bogdan I. Florea, Frank Heppner, Elke Krüger, Markus M. Rinschen, Oliver Kretz, Roland Thünauer, Catherine Meyer-Schwesinger

===

List of Content
Author Information

Supplementary Figures

Supplementary Figure 01. Glomerular cell-specific distribution of proteasomal and lysosomal proteins in human glomeruli.

Supplementary Figure 02. Glomerular cell-specific distribution of proteasomal and lysosomal proteins in murine glomeruli.

Supplementary Figure 03. Differential expression of proteasome transcripts and proteins in glomerular cell-types.

Supplementary Figure 04. Differential expression of transcripts to deubiquitinating enzymes (DUBs) and shuttling factors in human and murine glomerular cell-types

Supplementary Figure 05. Protein levels of common loading controls in glomerular cells

Supplementary Figure 06. Differential expression of the endo-lysosome transcripts and proteins in glomerular cell-types

Supplementary Figure 07. Cell cycle analysis of glomerular endothelial cells.

Supplementary Figure 08. Glomerular endothelial β5i-deficiency results in alterations of the filtration barrier.

Supplementary Figure 09. Successful glomerular proteasome and lysosome inhibition in mice

Supplementary Figure 10. Glomerular ultrastructure is unaffected in vehicle-treated control mice

Supplementary Figure 11. Proteasome impairment in podocytes is not compensated by the autophagosome lysosome pathway

Supplementary Figure 12. Proteasome inhibition results in mouse IgG deposition in the subepithelial space.

Supplementary Figure 13. Proteasome functionality affects podocyte endocytosis

Supplementary Figure 14. Fcγ receptor transcript and protein abundance in bulk-isolated glomerular cell types in dependence of proteasome functionality.

Supplementary Figure 15. Transcript and protein expression pattern of phagocytosis-related proteins in glomerular cell types.

Supplementary Figure 16. Expression pattern of differentially regulated endocytosis-related proteins in glomerular cell types.

Supplementary Table 01. RT-qPCR Primer sequences used in the study.

Author/Institute Information

Wiebke Sachs[1,2], Lukas Blume[1,2], Desiree Loreth[1,2], Lisa Schebsdat[1,2], Favian Hatje[1,2], Sybille Koehler[3,2], Uta Wedekind[1,2], Marlies Sachs[1,2], Stephanie Zieliniski[1,2], Johannes Brand[1,2], Christian Conze[4], Bogdan I. Florea[5], Frank Heppner[6], Elke Krüger[7], Markus M. Rinschen[3,2], Oliver Kretz[3,2], Roland Thünauer[4,8,9], Catherine Meyer-Schwesinger[1,2]

[1]Institute of Cellular and Integrative Physiology, Center for Experimental Medicine, University Medical Center Hamburg-Eppendorf, Hamburg, Germany

[2]Hamburg Center of Kidney Health, Hamburg, Germany

[3]Nephrology, III Medical Clinic, Department of Internal Medicine, University Medical Center Hamburg-Eppendorf, Hamburg Germany

[4]Leibniz Institute of Virology, Hamburg, Germany

[5]Bio-organic synthesis group, Leiden University, Leiden, the Netherlands

[6]Institute of Neuropathology, Charité, Berlin, Germany

[7]Institute of Medical Biochemistry and Molecular Biology, University Medicine Greifswald, Germany

[8]Technology Platform Light Microscopy (TPLM), University Hamburg, Germany

[9]Advanced Light and Fluorescence Microscopy (ALFM) facility at the Centre for Structural Systems Biology (CSSB), Hamburg, Germany

Suppl. Fig.1

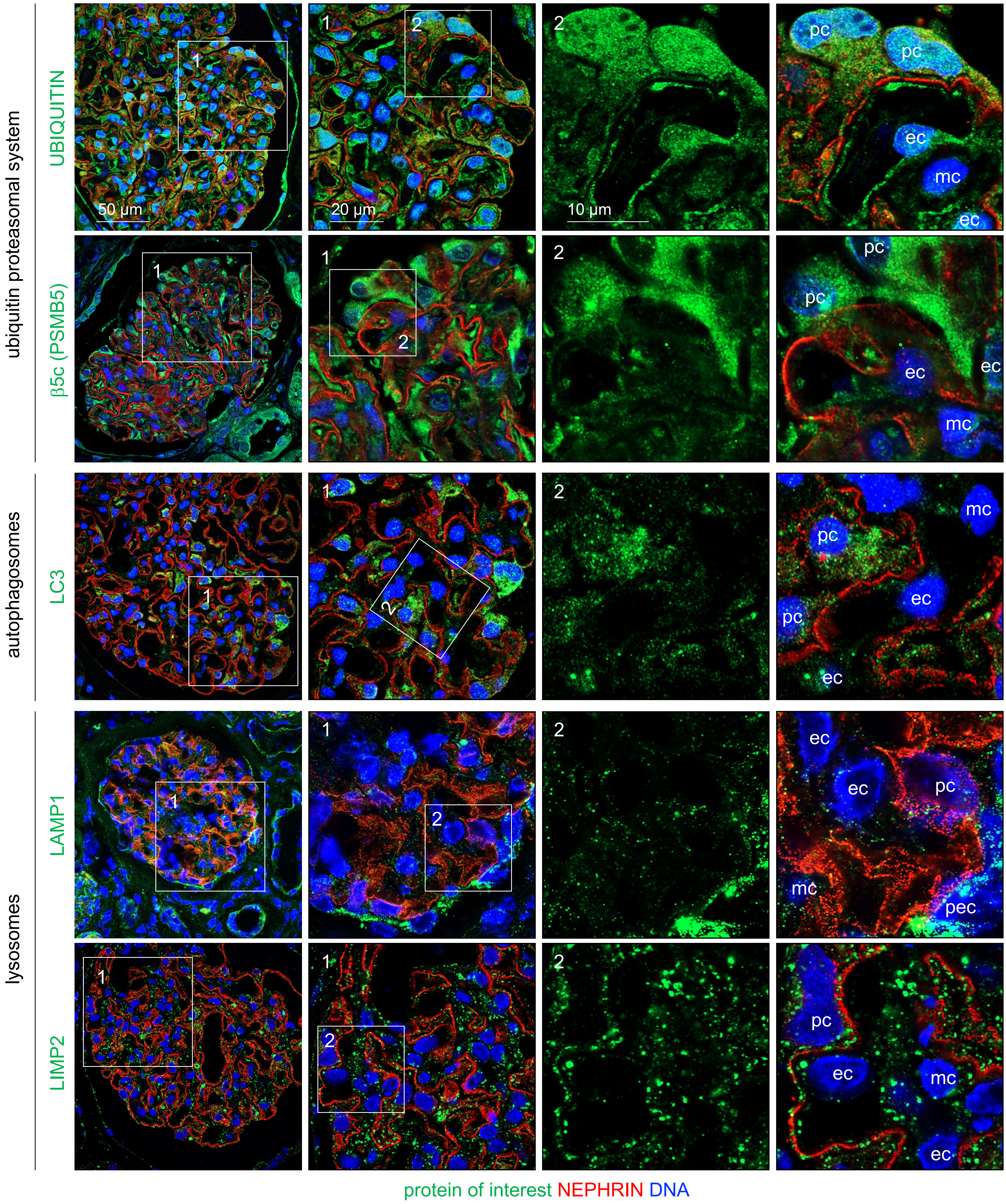

Glomerular cell-specific distribution of proteasomal and autophagosomal-lysosomal proteins in human glomeruli. The distribution of ubiquitin proteasomal and autophagosomal-lysosomal marker proteins were analyzed by high-resolution confocal images of human kidney from the healthy part of tumor nephrectomy samples. Micrographs were analyzed from 3 individual experiments, with 3 micrographs per group. Ubiquitin and the constitutive proteasomal subunit β5c (PSMB5) serve as markers for the ubiquitin proteasome system, the microtubule-associated protein 1A/1B-light chain 3 (LC3) as a marker of autophagosomes, and the lysosomal-associated membrane protein 1 (LAMP1) and the lysosomal integral membrane protein-2 (LIMP2) as lysosomal markers. The target protein is depicted in green, the slit membrane protein NEPHRIN to visualize the glomerular filtration barrier in red, and the DNA via Hoechst staining in blue; pc = podocyte, mc = mesangial cell, ec = glomerular endothelial cell, pec = parietal epithelial cell.

Suppl. Fig. 2

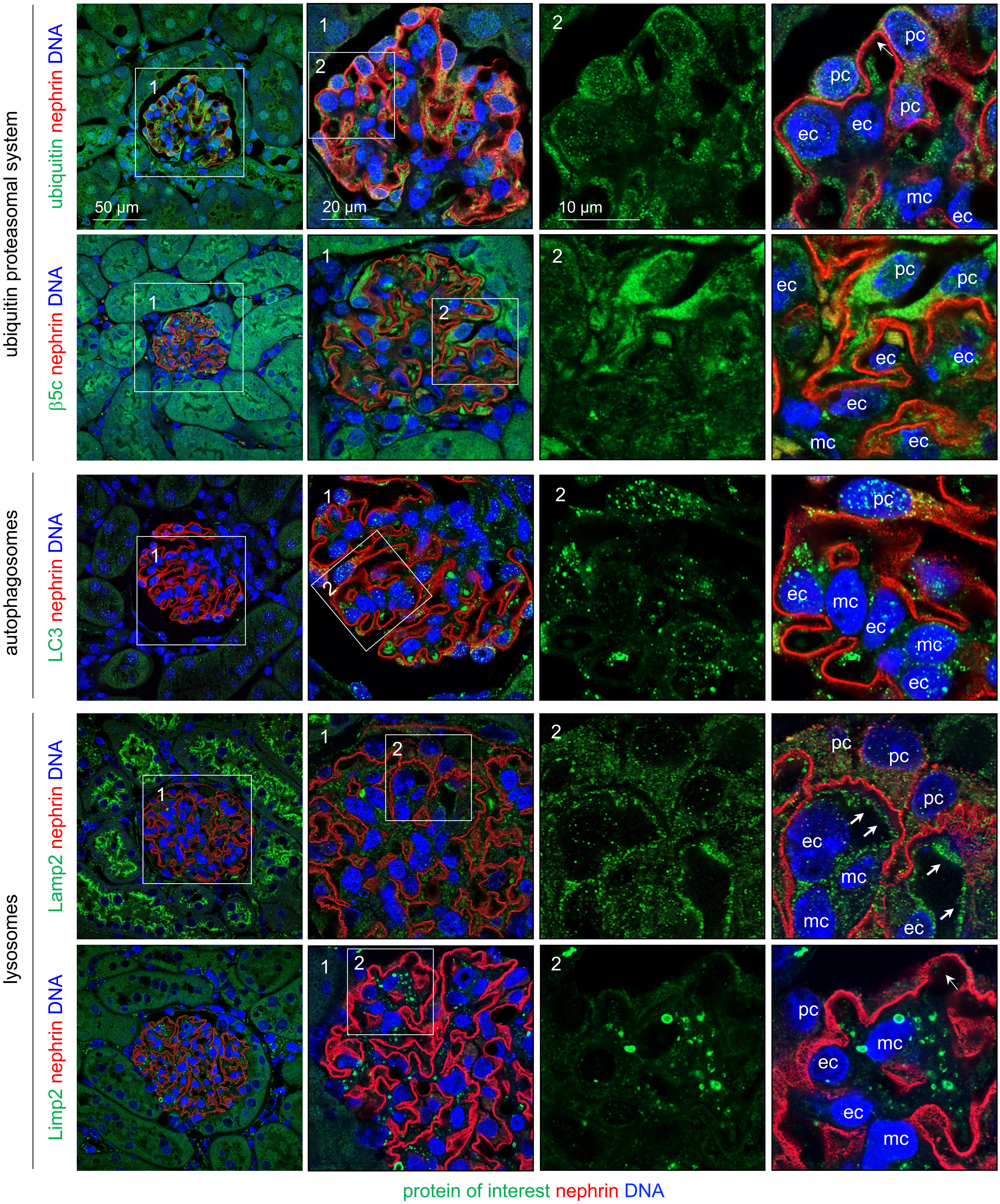

Glomerular cell-specific distribution of proteasomal and lysosomal proteins in murine glomeruli. Micrographs were analyzed from 3 individual experiments, with 3 micrographs per group. The distribution of ubiquitin proteasomal and autophagosomal-lysosomal marker proteins were analyzed by high-resolution confocal images of murine kidney. Ubiquitin and the constitutive proteasomal subunit β5c serve as markers for the ubiquitin proteasomal system, the microtubule-associated protein 1A/1B-light chain 3 (LC3) as a marker of autophagosomes, and the lysosomal-associated membrane protein 2 (Lamp2) and the lysosomal integral membrane protein-2 (Limp2) as lysosomal markers. The target protein is depicted in green, the slit membrane protein nephrin to visualize the glomerular filtration barrier in red, and the DNA via Hoechst staining in blue; pc = podocyte, mc = mesangial cell, ec = glomerular endothelial cell, arrows point towards endothelial lining filled with lamp2 positive lysosomes.

Suppl. Fig. 3

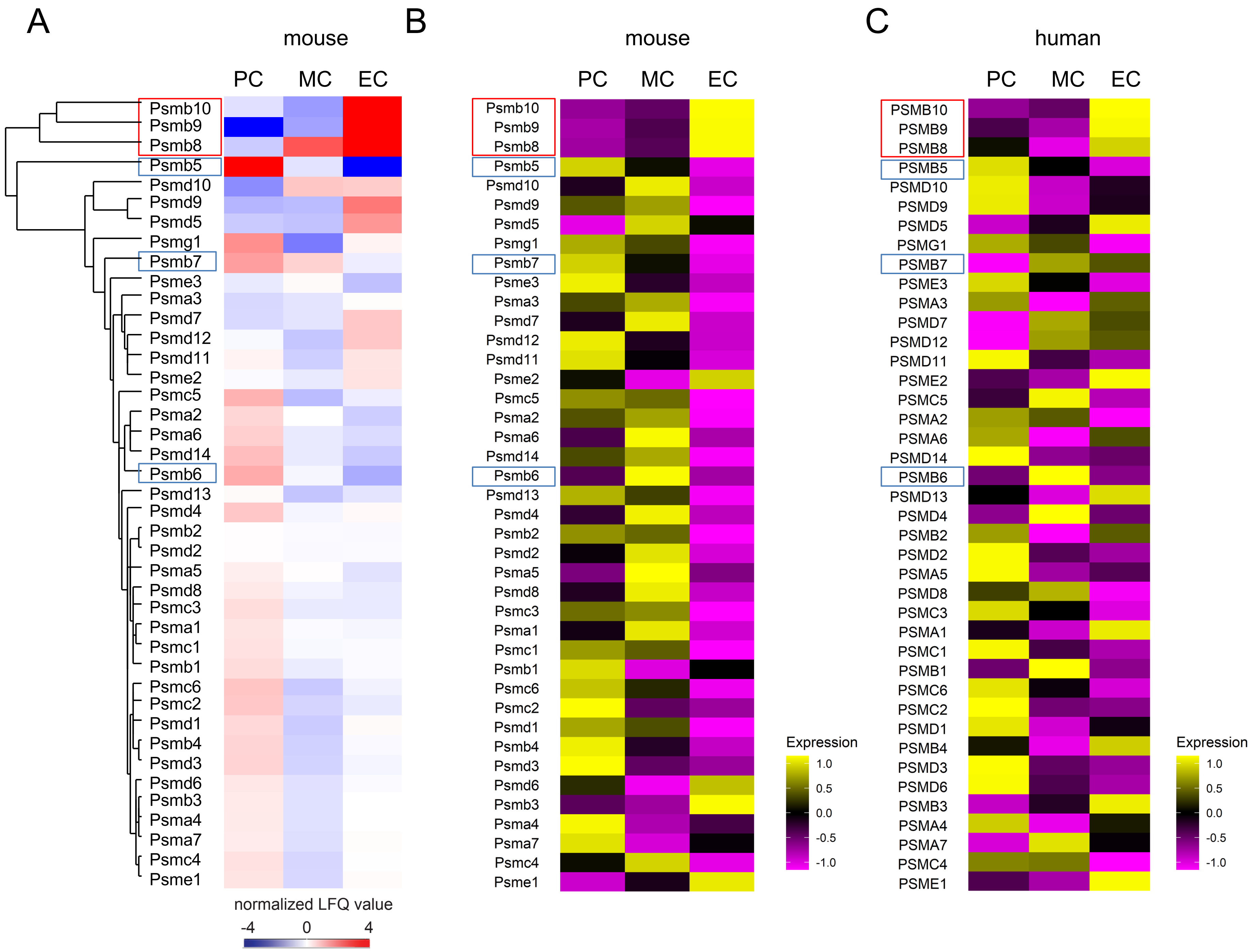

Differential expression of proteasome transcripts and proteins in glomerular cell-types. (**A**) Glomerular cells from naïve male BALB/c mice were bulk-separated by FACS-sort and analyzed for the expression of ubiquitin proteasome system proteins. Protein values were obtained by label-free quantification results using the MaxQuantLFQ algorithm https://pubmed.ncbi.nlm.nih.gov/24942700/. The heatmaps depict euclidian distance clustering of proteins associated with the proteasomal degradation system. PC = podocytes, MC = mesangial cells, EC = glomerular endothelial cells. The boxed transcripts/proteins represent the proteolytic constitutive proteasome subunits (blue) or the proteolytic immunoproteasome subunits (red). (**B**) Single cell transcripts of PC, MC, and GEnC derived from a published murine glomerular single cell RNAseq dataset (from He, B. et al. Nat Commun 2021 Vol. 12 Issue 1 Pages 2141) or (**C**) derived from human kidney single cell RNAseq data accessible within the Kidney Precision Medicine Project (KPMP, accessed 12/04/2023; https://www.kpmp.org) were analyzed for the expression of proteasome-related transcripts. Heatmaps depict the relative transcript levels of podocytes, glomerular endothelial and mesangial cells to total murine glomerular cell transcript levels (mouse) of the preparations or to normalized transcript levels of PC, MC, and GEnCs in the KPMP database.

Suppl. Fig. 4

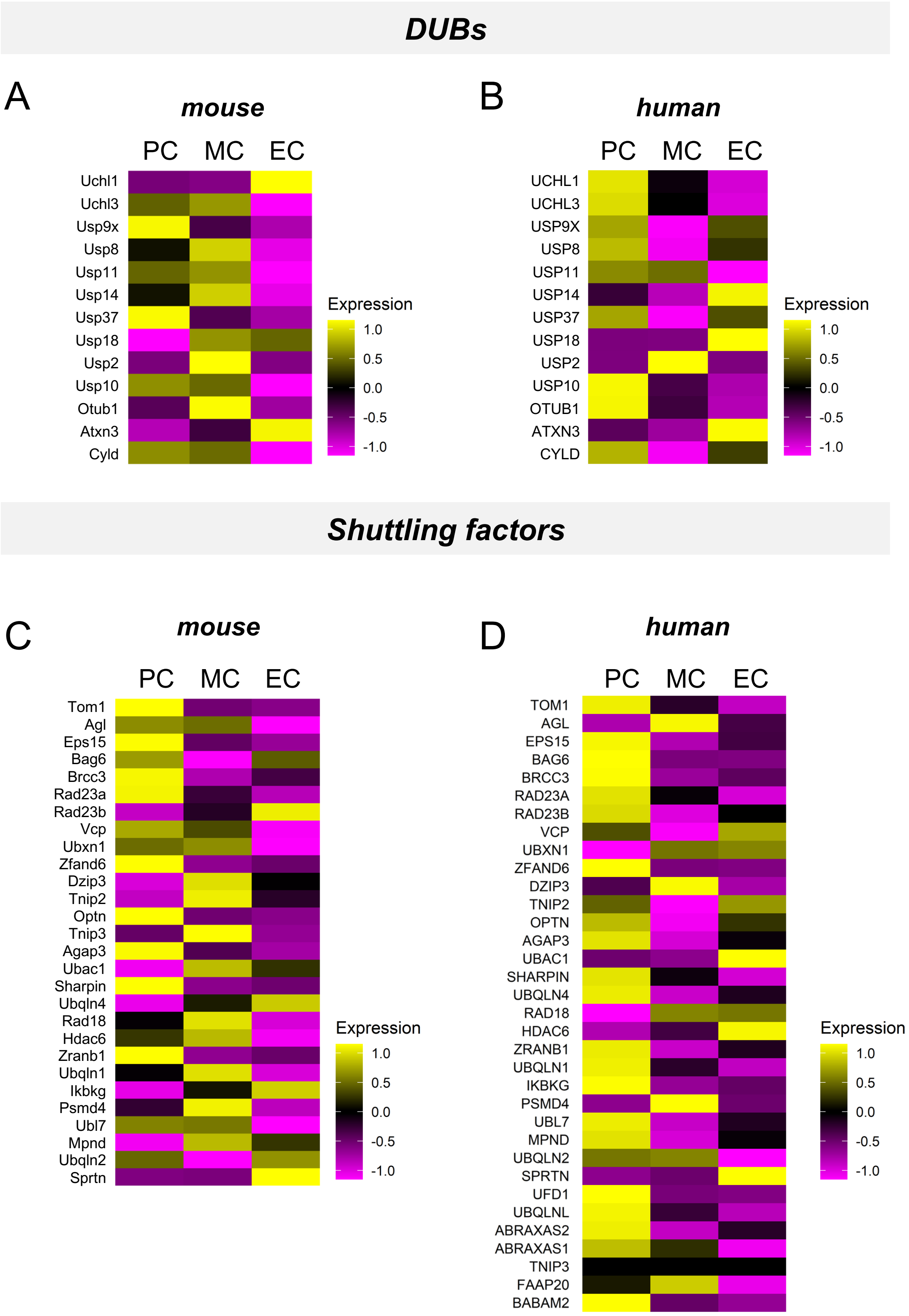

Differential expression of transcripts to deubiquitinating enzymes (DUBs) and shuttling factors in human and murine glomerular cell-types. Single cell RNAseq data sets from (**A, C**) mouse or (**B, D**) human single cells were analyzed for DUBs and shuttling factor transcripts (GO-Term GO:0031593 "polyubiquitin modification-dependent protein binding“). Murine single cell transcripts of PC, MC, and GEnC are derived from a published murine glomerular single cell RNAseq dataset (from He, B. et al. Nat Commun 2021 Vol. 12 Issue 1 Pages 2141). Human kidney single cell RNAseq data accessible within the Kidney Precision Medicine Project (KPMP, accessed 12/04/2023; https://www.kpmp.org) were used for analyses. Heatmaps depict the relative transcript levels of podocytes, glomerular endothelial and mesangial cells to total murine glomerular cell transcript levels (mouse) of the preparations or to normalized transcript levels of PC, MC, and GEnCs in the KPMP database.

Suppl. Fig. 5

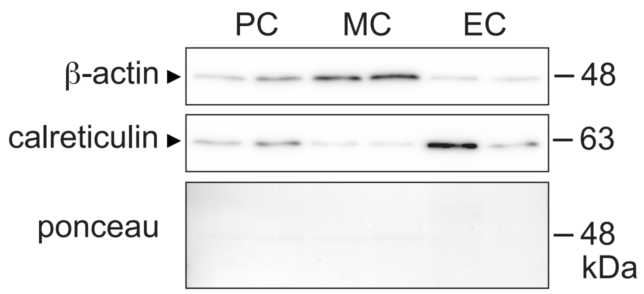

Protein levels of common loading controls in glomerular cells. Immunoblot of β-actin, calreticulin, and ponceau staining in bulk-isolated glomerular cells. PC = Podocytes, MC = mesangial cells, EC = glomerular endothelial cells. Equal cell numbers were loaded. Representative blot from 2 individual experiments, n = 5 mice. Source data are provided as a Source data file.

Suppl. Fig. 6

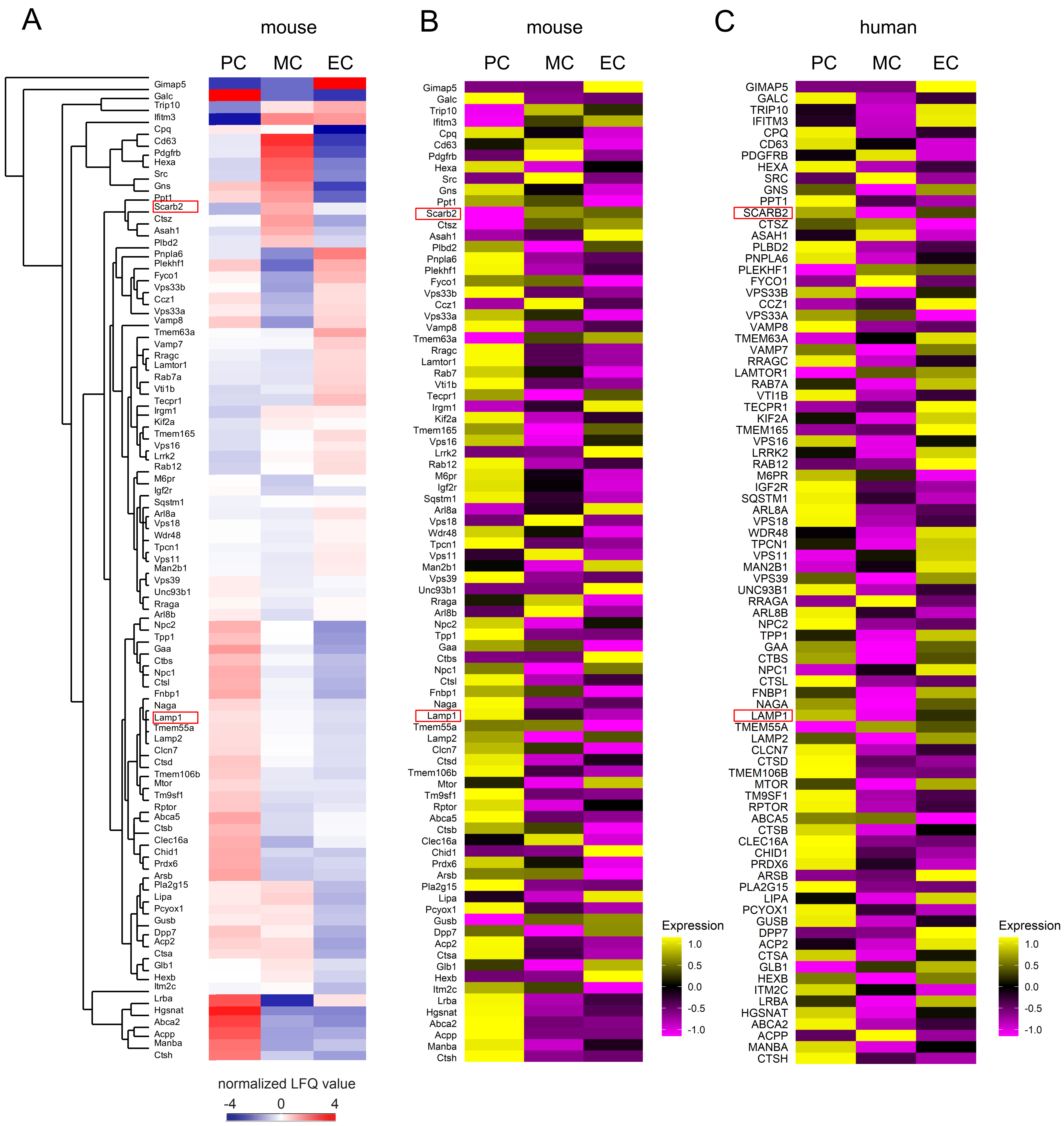

Differential expression of the endo-lysosome transcripts and proteins in glomerular cell-types. (**A**) Glomerular cells from naïve male BALB/c mice were bulk-separated by FACS-sort and analyzed for the expression of endo-lysosomal pathway proteins. Protein values were obtained by label-free quantification results using the MaxQuantLFQ algorithm https://pubmed.ncbi.nlm.nih.gov/24942700/. The heatmaps depict euclidian distance clustering of proteins associated with the endo-lysosomal degradation system, PC = podocytes, MC = mesangial cells, EC = glomerular endothelial cells. Red emboxed transcripts or proteins were also analyzed via qPCR, histology, or immunoblot. (**B**) Single cell transcripts of PC, MC, and GEnC derived from a published murine glomerular single cell RNAseq dataset (from He, B. et al. Nat Commun 2021 Vol. 12 Issue 1 Pages 2141) or (**C**) derived from human kidney single cell RNAseq data accessible within the Kidney Precision Medicine Project (KPMP, accessed 12/04/2023; https://www.kpmp.org) were analyzed for the expression of endosome-lysosome related transcripts. Heatmaps depict the relative transcript levels of podocytes, glomerular endothelial and mesangial cells to total murine glomerular cell transcript levels (mouse) of the preparations or to normalized transcript levels of PC, MC, and GEnCs in the KPMP database.

Suppl. Fig. 7

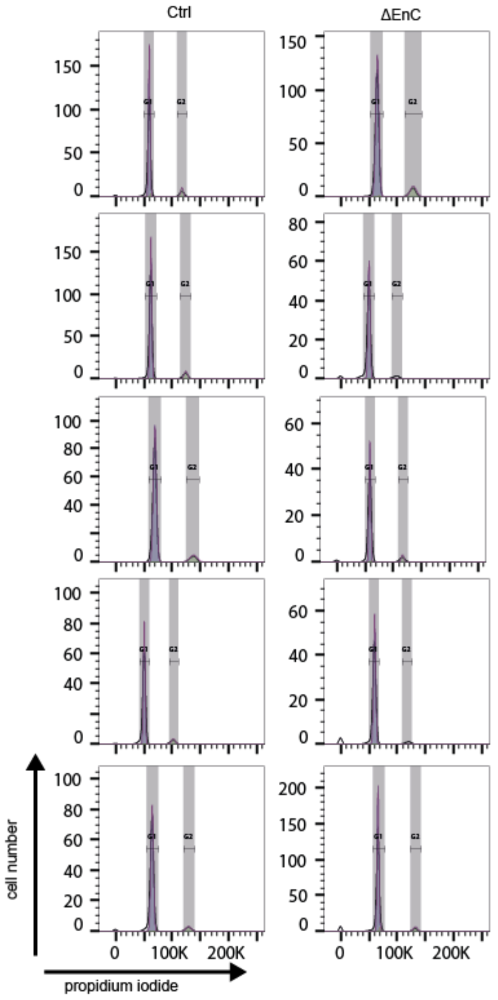

Cell Cycle analysis of glomerular endothelial cells: Flow cytometry plots of the propidium iodide staining of glomerular endothelial cells derived from *Lmp7*$^{\Delta EnC}$ mice and littermate controls.

Suppl. Fig. 8

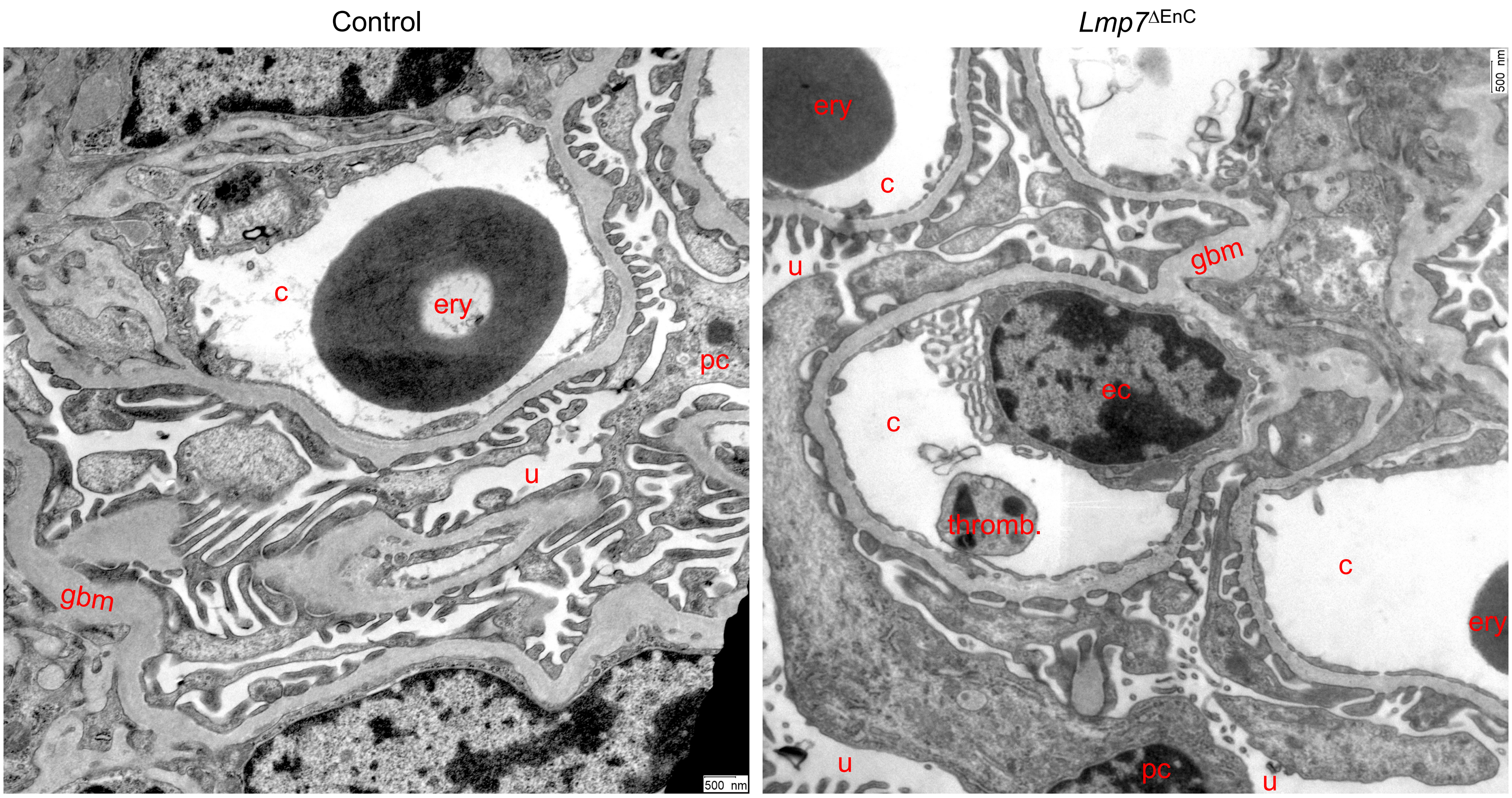

Glomerular endothelial β5i-deficiency results in alterations of the filtration barrier. Micrographs were analyzed from 1 individual experiment, with 3 micrographs per group. Overview of electron microscopical ultrastructural analyses shown in Fig. 3G. In the *Lmp7*$^{\Delta EnC}$ glomerulus, glomerular endothelial cell fenestrations are lost, podocyte foot processes are effaced, and the glomerular basement membrane exhibits focal splitting with irregularity in thickness; c = capillary lumen, u = urinary space, gbm = glomerular basement membrane, pc = podocyte, ec = endothelial cell, thromb = thrombocyte, ery = erythrocyte.

Suppl. Fig. 9

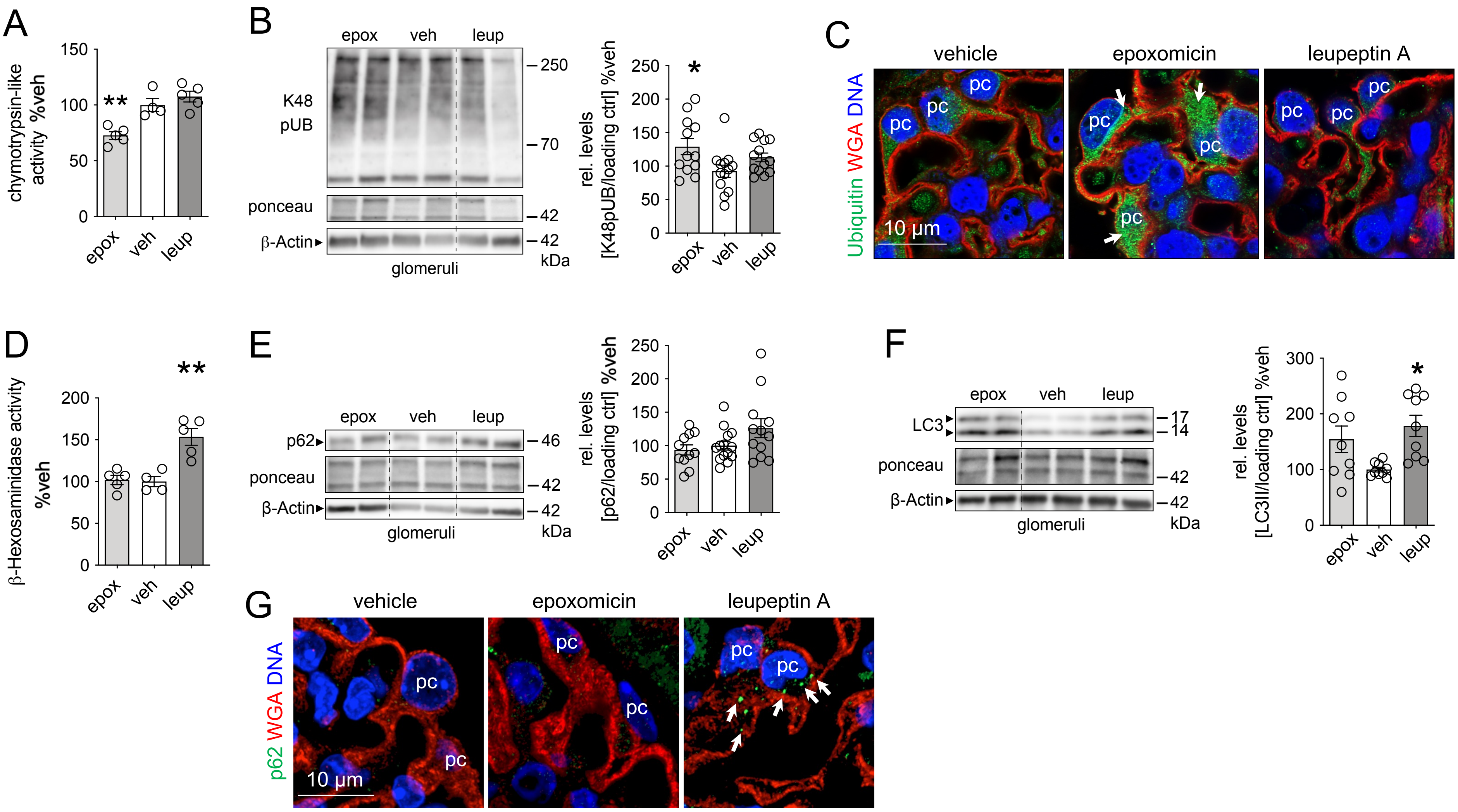

Successful glomerular proteasome and lysosome inhibition in mice. Naïve BALB/c males aged 14-20 weeks were treated with the irreversible proteasome inhibitor epoxomicin (epox, 0,5 µg/g bodyweight), the lysosome inhibitor leupeptin A (leup, 40 µg/g bodyweight) or equal volumes of DMSO (veh, vehicle, 125 µl) on four consecutive days. Thereafter, kidneys were collected, and glomeruli were isolated. (**A-C**) Epoxomicin application successfully impaired proteasome function. (**A**) To assess the main proteolytic activity of the proteasome, glomerular chymotrypsin-like activity was measured, data pooled from 1 experiment, $^{**}p = 0.0056$, mean ± SEM, n = 4 (epox), n = 5 (veh, leup) mice, One-Way ANOVA with Bonferroni´s post-test for multiple comparisons. (**B**) Immunoblot quantification of glomerular K48-polyubiquitinated protein abundance depicts accumulation of proteasome substrates in glomeruli of epoxomicin-treated mice, densitometric quantification, mean ± SEM, n = 12 (epox), n = 13 (veh, leup), mice pooled from 4 independent experiments, $^{*}p = 0.0301$, One-way ANOVA with Bonferroni's post-test for multiple comparisons. (**C**) High-resolution confocal micrographs of ubiquitinated protein (green) expression; wheat germ agglutinin (WGA, red) visualizes the general morphology by binding to N-acetyl-glucosamine and sialic acid, DNA is depicted in blue using Hoechst. Note the specific and accentuated enhancement of ubiquitin expression in podocytes (pc, arrows). (**D-G**) Leupeptin A application successfully impaired lysosome function. (**D**) To assess the activity of lysosomal enzymes the β-hexosaminidase activity was measured in isolated glomeruli, data pooled from 1 experiment, $^{**}p = 0.0017$, mean ± SEM, n = 4 (epox), n = 5 (veh, leup), mice One-Way ANOVA with Bonferroni´s post-test for multiple comparisons. (**E, F**) Immunoblot quantification of the ubiquitin receptor p62, n = 14 (veh), n = 11 (epox), n = 12 (leup) mice (**E**), which preferentially targets proteins to autophagosomes and of the autophagosome marker LC3, n = 9 mice per group, (**F**) in its membrane-bound lipidated (LC3-II, 14 kDa) and cytoplasmic (LC3-I, 17 kDa) form. Both, p62 as well as LC3-II accumulate in the setting of autophago-lysosomal impairment. Graph exhibits the densitometric quantification, mean ± SEM, $^{*}p = 0.0130$ pooled from 4 independent experiments, One-way ANOVA with Bonferroni's post-test for multiple comparisons. (**G**) High-resolution confocal micrographs of p62 (green) expression; wheat germ agglutinin (WGA, red) visualizes the general morphology by binding to N-acetyl-glucosamine and sialic acid, DNA is depicted in blue using Hoechst. Note the specific and accentuated enhancement of p62 aggregates (arrows) in podocytes (pc). Micrographs were analyzed from 3 individual experiments, with 3 micrographs per group. Source data are provided as a Source Data file.

Suppl. Fig. 10

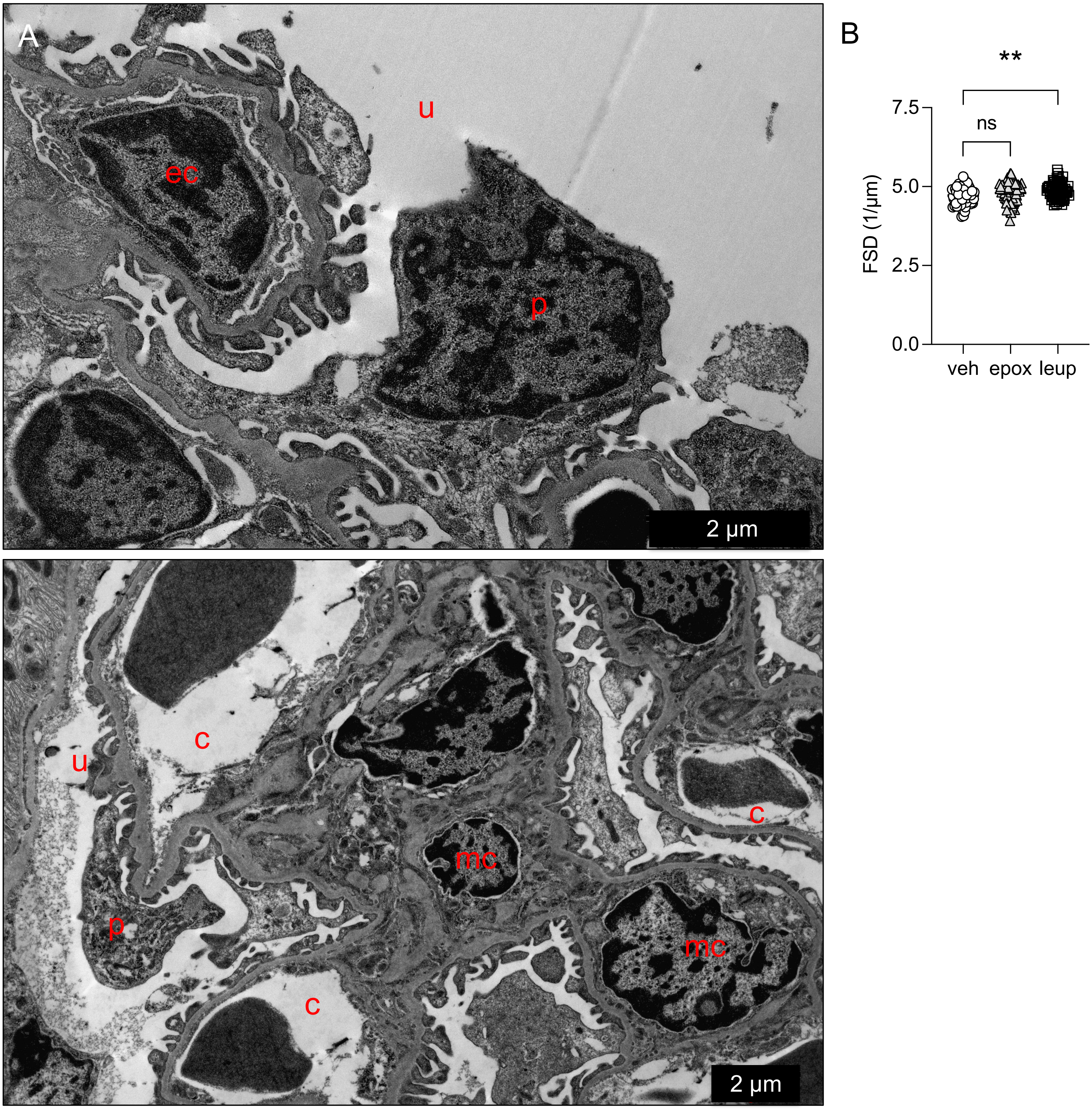

Glomerular ultrastructure is unaffected in vehicle-treated control mice. Naïve BALB/c males aged 14-20 weeks were treated with DMSO in PBS (vehicle, veh, 125 µl) on four consecutive days. Thereafter, kidneys were collected. Micrographs were analyzed from 3 individual experiments, with 3 micrographs per group. (**A**) Electron microscopic analyses exhibit normal ultrastructure of the glomerular filtration barrier and of the glomerular cells, c = capillary, u = urine, mc = mesangial cell, pc = podocyte, ec = glomerular endothelial cell. (**B**) Analysis of filtration slit density (FSD) in glomeruli from BALB/c males aged 14-20 weeks treated with either vehicle (DMSO in PBS), epoxomicin (epox) or leupeptin A (leup) on 4 consecutive days. The graph exhibits the FSD from three mice per condition. Statistical analysis: Mean ± SEM, ***p* = 0.0052, n = 3 mice, One-way ANOVA with Holm-Sidak's post-test for multiple comparisons. Source data are provided as a Source Data file.

Suppl. Fig. 11

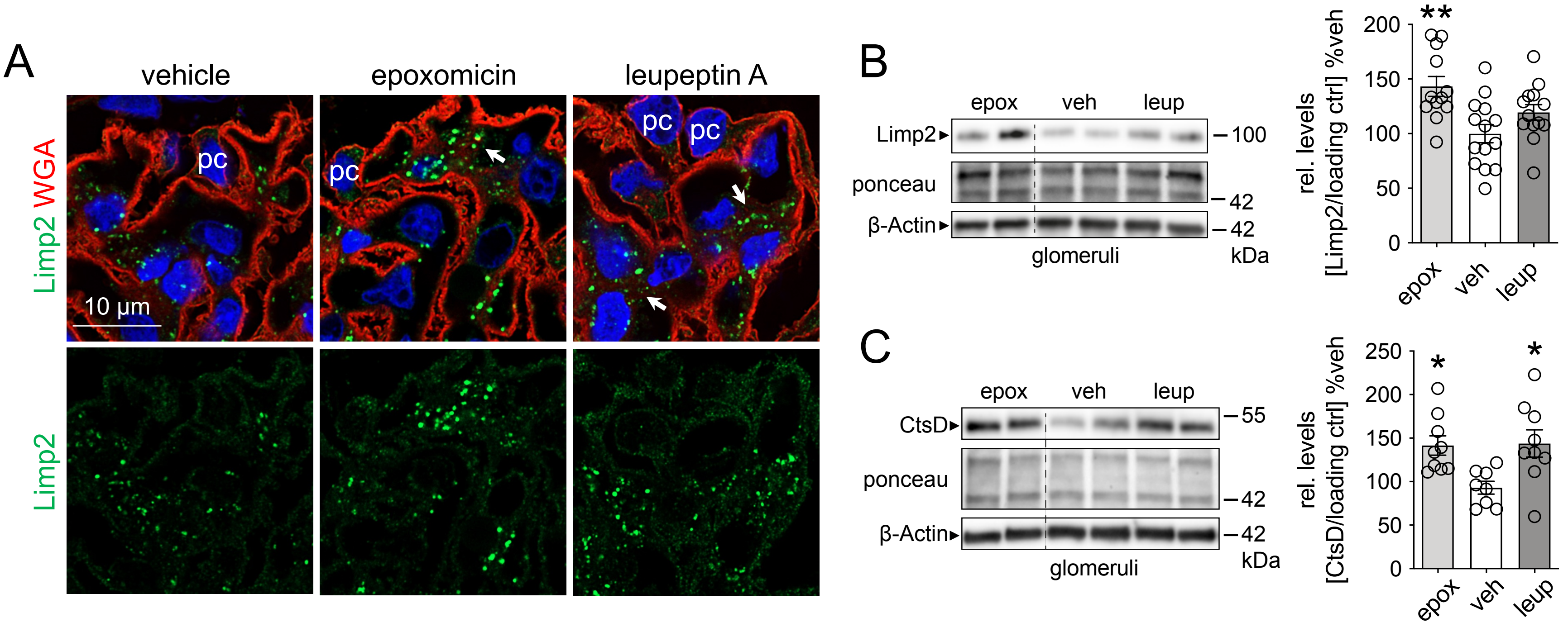

Proteasome impairment in podocytes is not compensated by the autophagosome-lysosome pathway. Naïve BALB/c males aged 14-20 weeks were treated with the irreversible proteasome inhibitor epoxomicin (epox, 0,5 µg/g bodyweight), the lysosome inhibitor leupeptin A (leup, 40 µg/g bodyweight) or equal volumes of DMSO (veh, vehicle, 125 µl) on four consecutive days. Thereafter, kidneys were collected, and glomeruli were isolated. Micrographs were analyzed from 3 individual experiments, with 3 micrographs per group. (**A**) High-resolution confocal micrographs of immunofluorescent stainings for Limp2 (lysosomal integral membrane protein 2, green) demonstrates increased abundance of enlarged Limp2-positive lysosomes (arrows) in the mesangium and in GEnCs of leupeptin A and epoxomicin-treated mice but not in podocytes (pc) of epoxomicin-treated mice albeit an altered proteostasis due to proteasome impairment (see Suppl. Fig. 9). (**B, C**) Immunoblot quantification of glomerular Limp2, n = 14 (veh), n = 12 (epox), n = 13 (leup) mice (**B**) and of the lysosomal protease Cathepsin D protein n = 8 (veh), n = 9 (epox, leup) mice, ***p* = 0.0021 (**C**) abundance depicts upregulation of lysosomes in glomeruli of epoxomicin-treated and of leupeptin A-treated mice, mean ± SEM, pooled from 2-3 independent experiments, One-way ANOVA with Bonferroni's post-test for multiple comparisons. Source data are provided as a Source Data file.

Suppl. Fig. 12

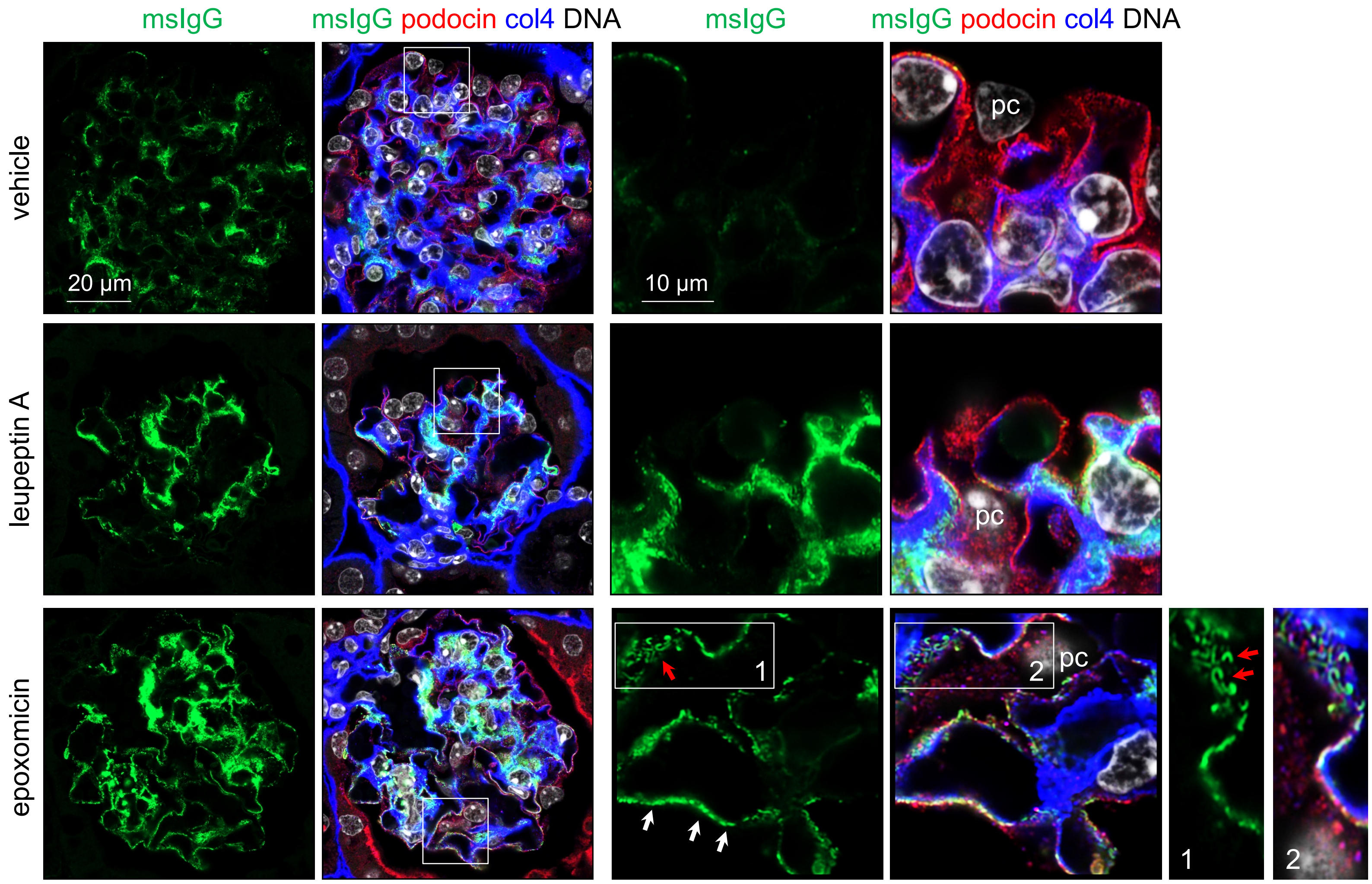

Proteasome inhibition results in mouse IgG deposition in the subepithelial space. Naïve BALB/c males aged 14-20 weeks were treated with the irreversible proteasome inhibitor epoxomicin (epox, 0,5 µg/g bodyweight), the lysosome inhibitor leupeptin A (leup, 40 µg/g bodyweight) or equal volumes of DMSO (veh, vehicle, 125 µl) on four consecutive days. Kidneys were removed on day 10. Micrographs were analyzed from 3 individual experiments, with 3 micrographs per group. High-resolution confocal micrographs depicting glomerular pattern of mouse IgG (msIgG, green) accumulation in inhibitor-treated mice. Arrows point towards linear (white arrows, due to frontal cut through the glomerular filtration barrier) and meandering (red arrows, due to horizontal cut through the glomerular filtration barrier) mouse IgG accumulations in the subepithelial space following proteasome inhibition. The glomerular basement membrane is marked by the structural protein collagen type 4 (blue), podocin (red) is used to demarcate podocyte (pc) foot processes, and DNA is depicted in white using Hoechst. Note the prominent msIgG accumulation in the mesangium after leupeptin A and epoxomicin treatment and the additional msIgG accumulation along the subepithelial space of the glomerular filtration barrier with accentuation at the slit membrane (meanders) only after epoxomicin treatment .

Suppl. Fig. 13

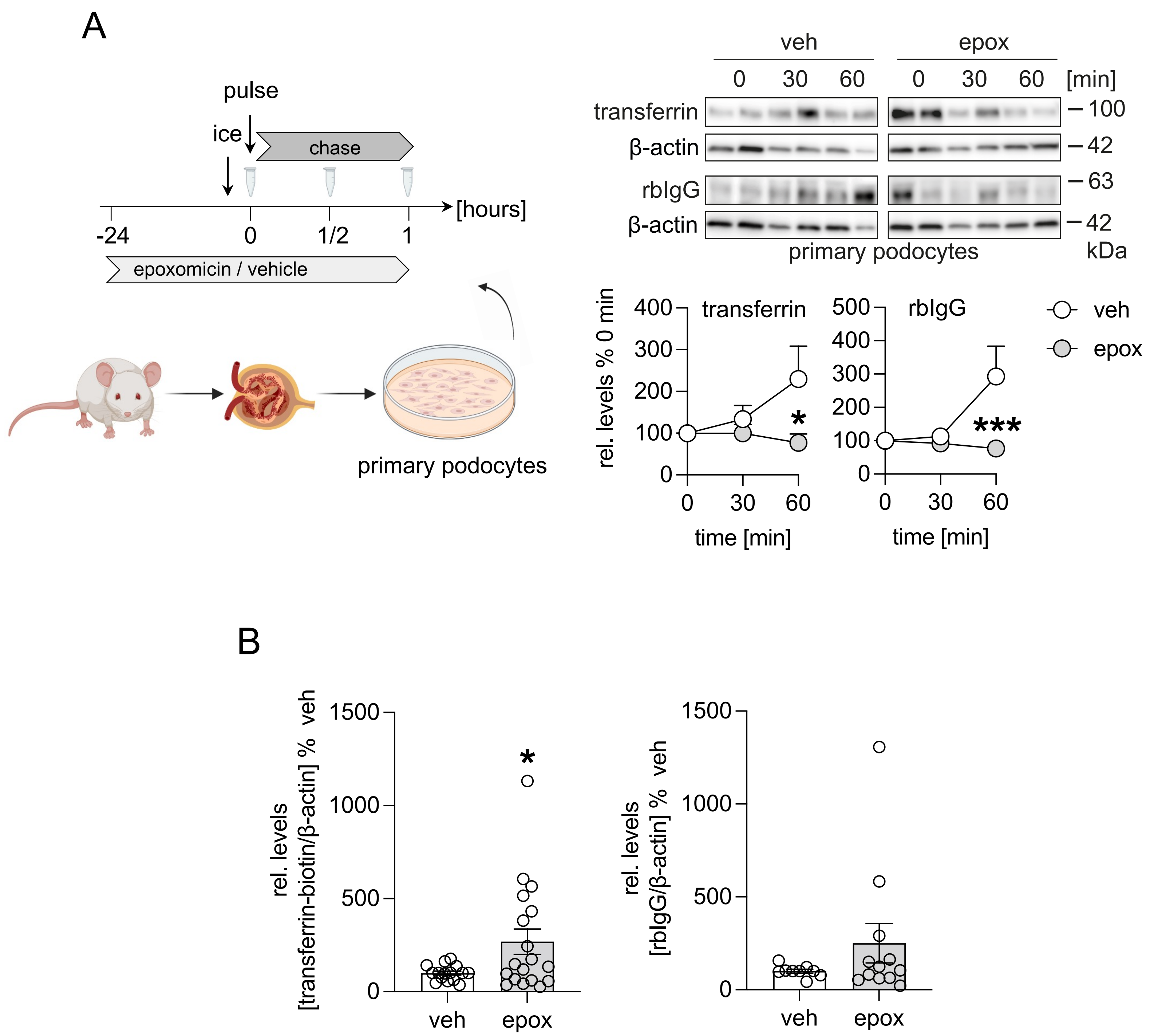

Proteasome functionality affects podocyte endocytosis. (**A**) Primary podocytes were outgrown from isolated decapsulated glomeruli for 5 days and assessed for their endocytic activity. Cells were pretreated with either 10 nM epoxomicin (epox) or an equivalent amount of DMSO (veh) for 24 h, synchronized on ice prior to the addition of endocytic substrates. Following extensive washing, cells were harvested after 0, 30, and 60 min and internalization of biotin-transferrin (clathrin-mediated) or rbIgG (clathrin-independent, FcRN-mediated) was assessed by immunoblotting, normalized to β-actin to control for equal loading. Biological replicates are shown. Values are expressed as relative levels to 0 h within the treatment groups, mean ± SEM, n = 12 per group, pooled data from 2 independent experiments, **p* = 0.0104, ****p* = 0.0006, Two-way ANOVA with Bonferroni's post-test for multiple comparisons. (**B**) Densitometric analysis of transferrin-biotin and rbIgG immunoblot of epoxomicin treated cells after 0 min of substrate exposure in relation to the amounts found in vehicle treated cells. Note the enhanced abundance of transferrin and rbIgG at time 0 in epoxomicin exposed primary podocytes, which indicates an increased adherence to the primary podocyte membrane. This could be either through unspecific binding ("stickiness") to the plasma membrane, or due to higher levels of receptors that bind the biotin-transferrin / rbIgG at the membrane and then fail to internalize. Values are expressed as relative to vehicle at 0 min, mean ± SEM, n = 15 (transferrin veh), n = 18 (transferrin epox), n = 9 (rbIgG veh), n = 12 (rbIgG epox), pooled data from 3 (transferrin) or 2 (rbIgG) independent experiments, **p* = 0.0331, unpaired t-test. Scheme was created with BioRender.com. Source data are provided as a Source Data file.

Suppl. Fig. 14

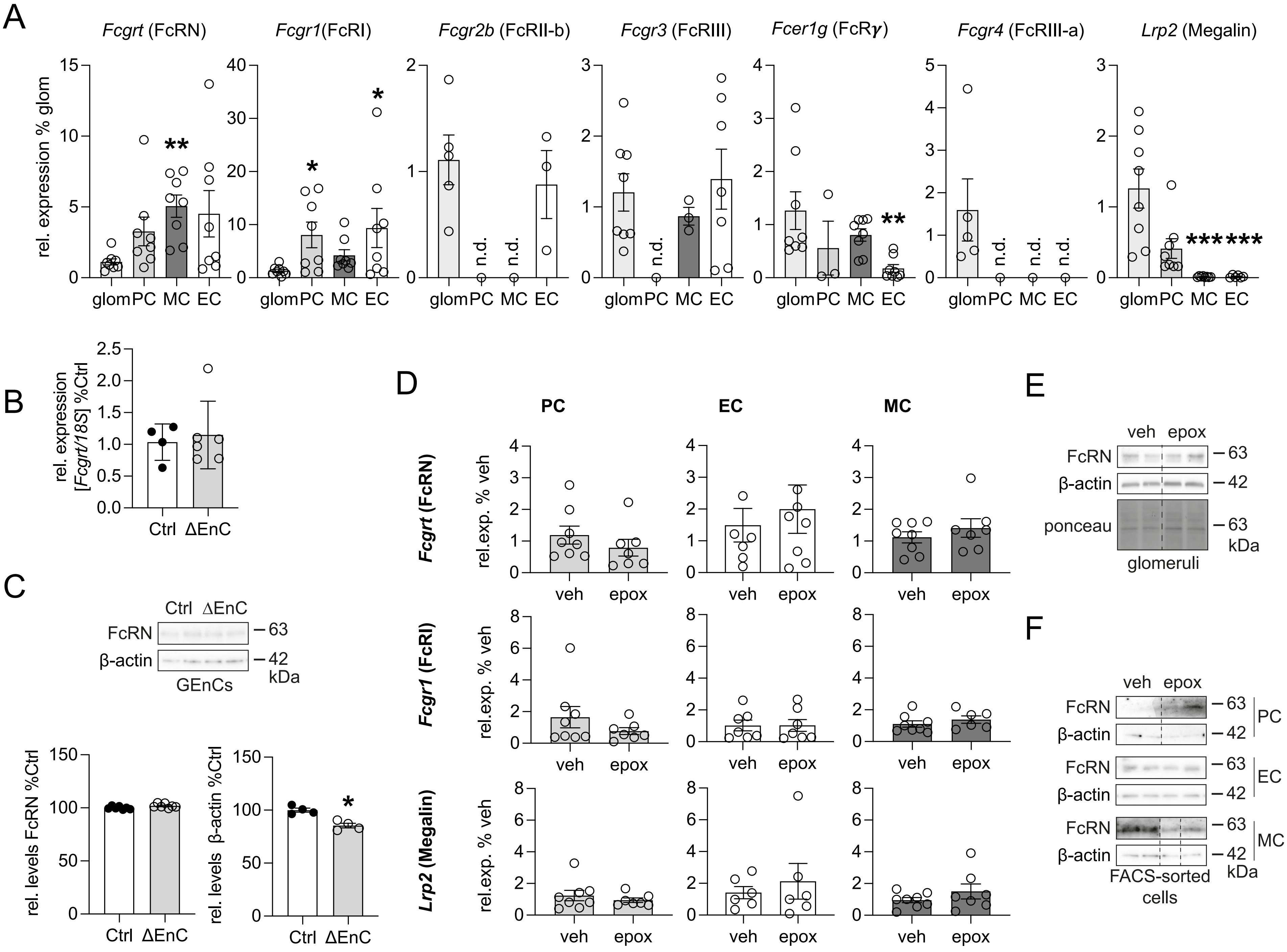

Fcγ receptor transcript and protein abundance in bulk-isolated glomerular cell types in dependence of proteasome functionality. Podocytes (PC), glomerular endothelial cells (GEnCs, EC), and mesangial cells (MC) were bulk-isolated from glomeruli (glom) of BALB/c mice aged 14-20 weeks in the naïve and proteasome-modified setting. Fcγ receptor transcript and protein abundance were quantified. (**A**) Relative expression of Fcγ receptor transcripts in naïve glomerular cell types to respective glomerular transcript levels determined by qRT-PCR, mean ± SEM, *Fcgrt* **p = 0.0034; *Fcgr1* PC *p = 0.0292, EC *p = 0.0494, *Fcer1g* **p = 0.0044, *Lrp2* MC ***p = 0.0002, EC ***p = 0.0004, n = 8 (*Fcgrt*, *Fcgr1*, *Fcgr3* (glom, PC), *Fcer1g* (glom, EC, MC), *Lrp2* (glom, PC, MC)), n = 5 (*Fcgr2b* (gloms, PC, MC), *Fcgr4* (gloms, PC, MC)), n = 3 (*Fcgr2b* (EC), *Fcgr3* (MC), *Fcer1g* (PC)), n = 7 (*Fcgr3* EC), n = 6 (*Lrp2* EC) from mice of 3 independent cell isolations, One-way ANOVA with Dunn‘s post-test for multiple comparisons; n.d. = non detected. (**B, C**) Comparative analyses of FcRN transcript and protein expression in GEnCs isolated from *Lmp7*$^{\Delta EnC}$ (ΔEnC) and control (Ctrl) littermates 5 weeks after knockout induction. (**B**) Relative *Fcgrt* expression determined by qRT-PCR normalized to 18S as home keeper, mean ± SEM, pooled data from 2 independent cell isolations, n = 4 Ctrl, n = 6 *Lmp7*$^{\Delta EnC}$ mice, no significance, Mann-Whitney-U test. (**C**) Immunoblot analysis of FcRN protein abundance in GEnCs loaded in a cell-number adapted manner between genotypes. Note the regulation of β-actin in β5i-deficiency, hence FcRN quantification between genotypes was not normalized to β-actin, mean ± SEM, pooled data from 2 independent cell isolations, n = 6 mice (FcRN), n = 4 (β-actin), *p = 0.0286 Mann-Whitney-U test. (**D-F**) Comparative analyses in glomerular cell types isolated from mice following a 4-day treatment with epoxomicin (epox, 0.5 µg/g b.w.) in relation to vehicle (veh, DMSO, 125 µl). (**D**) Relative mRNA expression to vehicle treatment of the most abundant Fcγ receptor transcripts in a cell type specific manner normalized to 18S as home keeper, mean ± SEM, pooled data from 3 independent cell isolations, n = 8 (veh PC and MC *Fcgrt*, *Fcgr1*, *Lrp2*; epox EC *Fcgrt*); n = 7 (epox PC *Fcgrt*, *Fcgr1*, *Lrp2*; veh EC *Fcgrt*, *Fcgr1*; epox EC *Fcgr1*; epox MC *Fcgrt*, *Lrp2*); n = 6 (EC *Lrp2*; epox MC *Fcgr1*) mice, no significance, Mann-Whitney-U test. (**E, F**) Immunoblot analysis of FcRN protein abundance in isolated glomeruli (**E**) and in glomerular cell types loaded in a cell-number adapted manner (**F**). Source data are provided as a Source Data file.

Suppl. Fig. 15

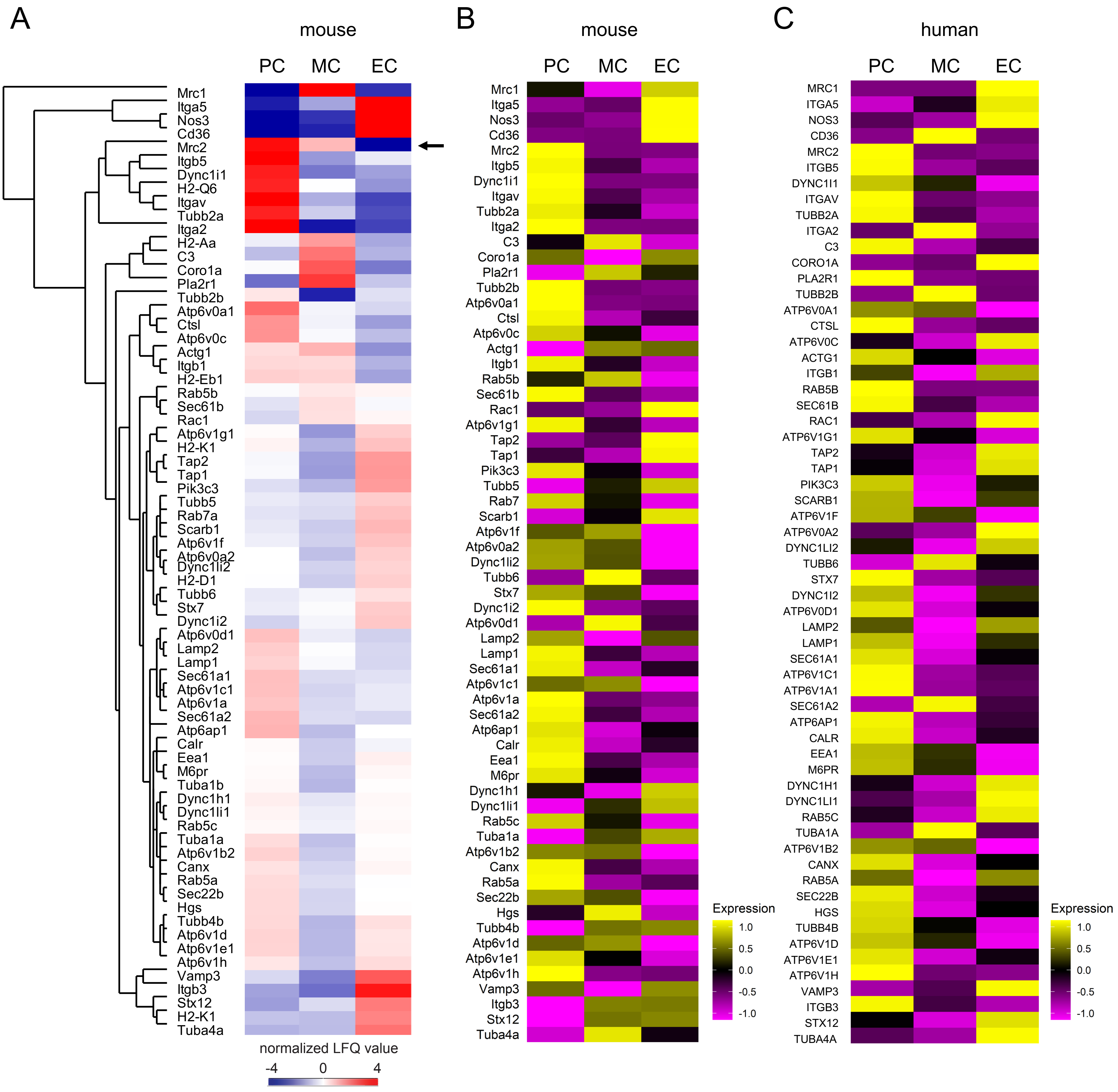

Transcript and protein expression pattern of phagocytosis-related proteins in glomerular cell types. (**A**) Glomerular cells from naïve male BALB/c mice were bulk-separated by FACS-sort and analyzed for the expression of phagocytosis pathway proteins. Protein values were obtained by label-free quantification results using the MaxQuantLFQ algorithm https://pubmed.ncbi.nlm.nih.gov/24942700/. The heatmaps depict euclidian distance clustering of proteins associated with phagocytosis, PC = podocytes, MC = mesangial cells, EC = glomerular endothelial cells. Arrow points towards Mrc2, which was chosen for subsequent analyses. (**B**) Single cell transcripts of PC, MC, and GEnC derived from a published murine glomerular single cell RNAseq dataset (from He, B. et al. Nat Commun 2021 Vol. 12 Issue 1 Pages 2141) or (**C**) derived from human kidney single cell RNAseq data accessible within the Kidney Precision Medicine Project (KPMP, accessed 12/04/2023; https://www.kpmp.org) were analyzed for the expression of phagocytosis-related transcripts. Heatmaps depict the relative transcript levels of podocytes, glomerular endothelial and mesangial cells to total murine glomerular cell transcript levels (mouse) of the preparations or to normalized transcript levels of PC, MC, and GEnCs in the KPMP database.

Suppl. Fig. 16

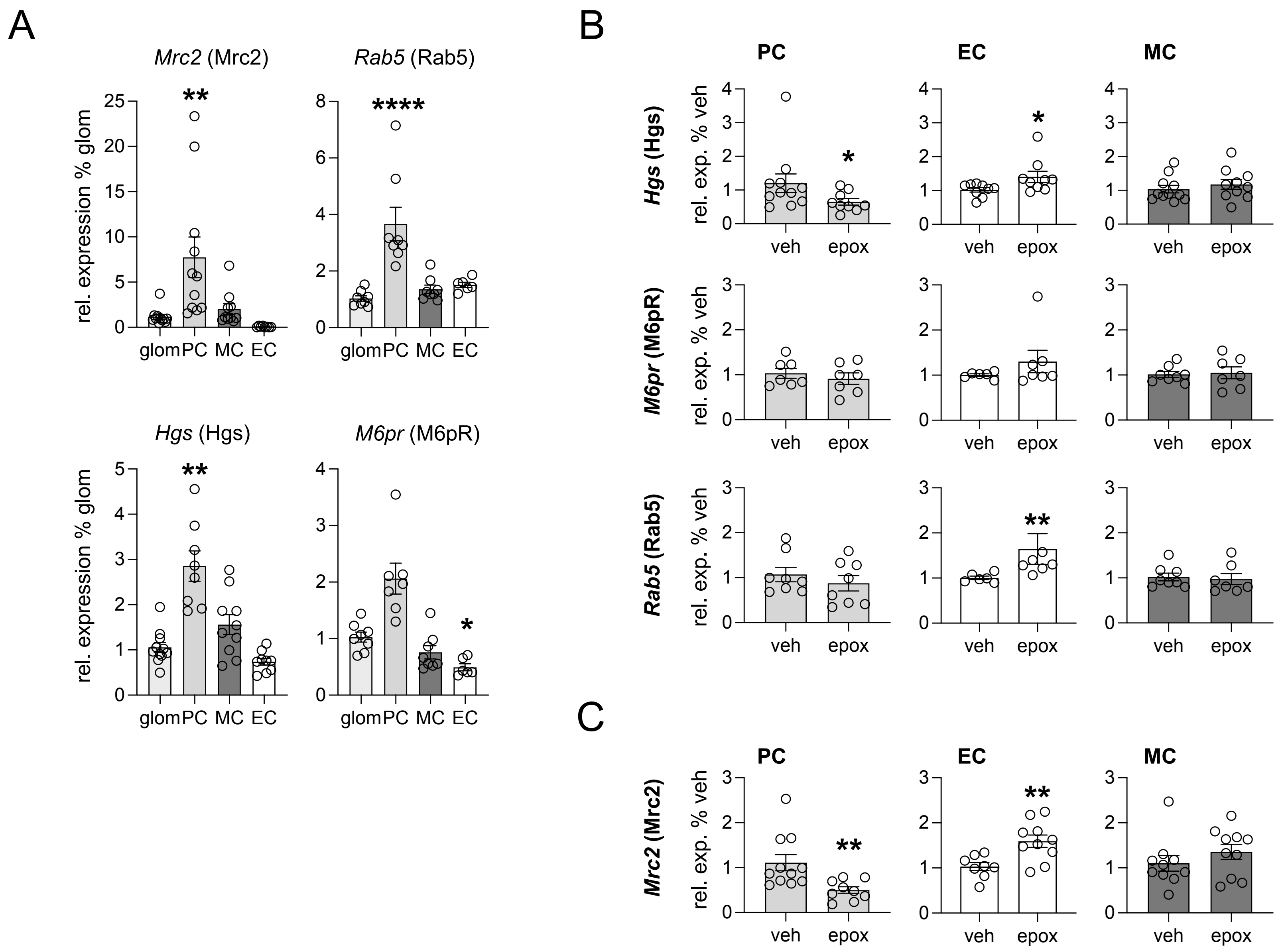

Expression pattern of differentially regulated endocytosis-related proteins in glomerular cell types. (**A**) Relative expression levels of *Mrc2*, *Hgs*, *M6pr* and *Rab5a* transcripts in glomerular cell-types analyzed *via* qRT-PCR in relation to the expression in glomeruli. Mean ± SEM, *Mrc2* **p = 0.0017, *Rab5* ****p < 0.0001, *Hgs* **p = 0.0031, *M6pr* *p = 0.0326, n = 11 (glom, PC *Mrc2*; glom *Hgs*), n = 8 (EC *Mrc2*; glom, PC, MC *Rab5*; PC *Hgs*; glom, MC *M6pr*), n = 10 (MC *Mrc2*, *Hgs*), n = 6 (EC *Rab5*, *M6pr*), n = 9 (EC *Hgs*), n = 7 (PC *M6pr*) mice, One-way ANOVA with Dunnett's post-test for multiple comparisons. (**B**) Relative expression levels of *Hgs, M6pr* and *Rab5a* determined by qRT-PCR in a cell-type specific manner after the treatment of naïve BALB/c mice with epoxomicin (epox, 0.5 µg/g bodyweight) in relation to vehicle (veh, DMSO, 125 µl) treated mice. Mean ± SEM, *Hgs* PC *p = 0.0465, *Hgs* EC *p = 0.0315, *Rab5* EC **p = 0.0047, n = 11 (veh PC, veh MC *Hgs*), n = 10 (epox MC *Hgs*), n = 9 (epox PC, EC *Hgs*), n = 8 (veh MC *M6pr*, PC *Rab5*; veh MC *Rab5*), n = 7 (PC *M6pr*; epox EC, MC *M6pr*; epox EC, MC *Rab5*), n = 6 (veh EC *M6pr*, *Rab5*) mice. Mann-Whitney-U test. (**C**) Relative *Mrc2* expression pattern in bulk-isolated podocytes (PC), GEnCs (EC), and mesangial cells (MC) of epoxomicin-treated mice compared to vehicle (DMSO) quantified *via* qRT-PCR, 18S was used as home keeper, mean ± SEM, n = 11 (veh PC), n = 10 (epox EC; MC), n = 9 (epox PC), n = 8 (veh EC), mice pooled data from 3 independent experiments, PC **p = 0.0016, EC **p = 0.0062 Mann-Whitney U test. Source data are provided as a Source Data file.

Suppl. Table 1

**Target Gene** (mouse)	**Forward Sequence** (5‘→ 3‘)	**Reverse Sequence** (5‘→ 3‘)
Psmb5	TAAGCATACACGGAGCCAGA	TGTGGCTGGGATAAGAGAGG
Psmb8	ACCAAAGGACCTCAGGAATG	GGACCCGGGACACTACAGTT
Scarb2	TTCACGTGGCTGGCGTGTCG	AGCTCAGGCAACAGGTAAGTCCCA
18S	TTCGAACGTCTGCCCTATCAA	CTGCCTTCCTTGGATGTGGTA
Mrc2	GCAGTGGCCATTGGAACATC	CGGCTTTCCGTGTGAGTTTC
Rab5a	GGCGCATCCGATCTCCG	AACTTCCAGGATGCAAGGCA
Hgs	CGCGTCAGCTCCGAAAGA	GACTCCCAGTCTGTCTCCAAC
M6pr	GAGTCAAAGAACGAGGTGGCT	CTGTATGTGTCTGAGCCCTGG
Fcgrt	ATATTAAATGGTCAGAAGAGGGGG	CCTCACCATTGAGGGCAAAC
Fcgr1	GCAAGTTAGAAGCGATGGCG	GTCCACAGTCACCCACTGAG
Fcgr2b	CCATCTGGACTGGAGCCAAC	GGCTTCGGGATGCTTGAGAA
Fcgr3	GCTGCTGTTTGCTTTTGCAG	GTCCCTTCGCACATCAGTGT
Fcer1g	AAGATCCAGGTCCGAAAGGC	TCTGAAGCTACTGGGGTGGT
Fcgr4	GCAGCTACCCTCCCAAGAAG	AGCAATGGCTAAGCACGGAA
Lrp2	GGCATAGGCCTCAGTGTTGT	GGGAAGGTAGGCACGTTTGA

qRT-PCR primer sequences used in the study.
