# Supplementary material for: The proteasome modulates endocytosis specifically in glomerular cells to promote kidney filtration
Source: Nat Commun. 2024 Mar 1;15:1897. doi: 10.1038/s41467-024-46273-0 (PMC10907641; doi:10.1038/s41467-024-46273-0)
Supplement: Supplementary file 3 — Description of Additional Supplementary Files [file 41467_2024_46273_MOESM3_ESM.pdf]

Description of Additional Supplementary Files

File name: Supplementary Movie 1

Description: Human podocytes were preincubated for 6 hours with DMSO as vehicle. Wheat germ agglutinin (WGA)-rhodamine in red was applied at timepoint 0 min to the medium to mark glycoproteins of the plasma membrane. Time-lapse live-cell images were taken for 50 min. Note the appearance of small motile WGA-positive vesicles and the motility of the plasma membrane. The WGA signal at the cell border decreases during the time-lapse.

File name: Supplementary Movie 2

Description: Human podocytes were preincubated for 6 hours with epoxomicin dissolved in DMSO. Wheat germ agglutinin (WGA)-rhodamine in red was applied at timepoint 0 min to the medium to mark glycoproteins of the plasma membrane. Time-lapse live-cell images were taken for 50 min. Note the reduced appearance of WGA-positive vesicles in epoxomicin pretreatment and the reduced motility of the plasma membrane. The WGA signal at the cell border is maintained during the time-lapse. Additionally, the large vesicles (white arrows) depicted in the epoxomicin treated cell exhibit a reduced motility.
