# Supplementary material for: The proteasome modulates endocytosis specifically in glomerular cells to promote kidney filtration
Source: Nat Commun. 2024 Mar 1;15:1897. doi: 10.1038/s41467-024-46273-0 (PMC10907641; doi:10.1038/s41467-024-46273-0)
Supplement: Supplementary file 6 — Reporting Summary [file 41467_2024_46273_MOESM6_ESM.pdf]

nature portfolio

Corresponding author(s): Catherine Meyer-Schwesinger

Last updated by author(s): Feb 2, 2024

Reporting Summary

Nature Portfolio wishes to improve the reproducibility of the work that we publish. This form provides structure for consistency and transparency in reporting. For further information on Nature Portfolio policies, see our Editorial Policies and the Editorial Policy Checklist.

Statistics

For all statistical analyses, confirm that the following items are present in the figure legend, table legend, main text, or Methods section.

n/a	Confirmed	
☐	☒	The exact sample size (*n*) for each experimental group/condition, given as a discrete number and unit of measurement
☐	☒	A statement on whether measurements were taken from distinct samples or whether the same sample was measured repeatedly
☐	☒	The statistical test(s) used AND whether they are one- or two-sided<br>*Only common tests should be described solely by name; describe more complex techniques in the Methods section.*
☒	☐	A description of all covariates tested
☐	☒	A description of any assumptions or corrections, such as tests of normality and adjustment for multiple comparisons
☐	☒	A full description of the statistical parameters including central tendency (e.g. means) or other basic estimates (e.g. regression coefficient) AND variation (e.g. standard deviation) or associated estimates of uncertainty (e.g. confidence intervals)
☐	☒	For null hypothesis testing, the test statistic (e.g. *F*, *t*, *r*) with confidence intervals, effect sizes, degrees of freedom and *P* value noted<br>*Give P values as exact values whenever suitable.*
☒	☐	For Bayesian analysis, information on the choice of priors and Markov chain Monte Carlo settings
☒	☐	For hierarchical and complex designs, identification of the appropriate level for tests and full reporting of outcomes
☒	☐	Estimates of effect sizes (e.g. Cohen's *d*, Pearson's *r*), indicating how they were calculated

Our web collection on statistics for biologists contains articles on many of the points above.

Software and code

Policy information about availability of computer code

Data collection	Data collection was performed with<br>Fusion FX7EDGE V0.7 Imager (Vilbert Lourmat)<br>Amersham ImageQuant 600 or 800 (GE Healthcare, Cytiva)<br>ZEISS LSM800 with airyscan1 microscope<br>ZEISS LSM980 Airyscan 2 microscope<br>Visitron-SD-TIRF (with SoRa unit form Yokogawa)<br>Nikon Ti2 based Spinning Disc microscope equipped with a Yokogawa CSU-W unit<br>Andor iXON888 EMCCD camera (Oxford Instruments)<br>transmission-electron microscope (TEM 910)<br>QuantStudio 5 Real Time PCR System<br>Mithras LB 940<br>microplate spectrophotometer (BioTek, EL 808)<br>Autoanalyzer Hitachi 717, Roche<br>Aria IIIu or the BD FACS Fusion sorter (Becton Dickinson)<br>FACS Symphony A3 (BD) flow cytometer
Data analysis	Data analysis was performed with<br>GraphPad Prism, Version 9.5.0<br>Adobe Photoshop Version 23.0.1<br>Excel Version 16.68<br>Bio 1D Software (Vilbert Lourmat)

EvolutionCapt Software (Vilber Lourmat)
ZEN 3.6
ZEN 3.0
FlowJo 10.9.0
FIJI Version 2.0
ImageJ, version 1.53t.
R 4.3.2 (R Core Team (2023)
Seurat V5
MaxQuant and the LFQ algorithm
Perseus v 1.5.5.3

For manuscripts utilizing custom algorithms or software that are central to the research but not yet described in published literature, software must be made available to editors and reviewers. We strongly encourage code deposition in a community repository (e.g. GitHub). See the Nature Portfolio guidelines for submitting code & software for further information.

Data

Policy information about availability of data

All manuscripts must include a data availability statement. This statement should provide the following information, where applicable:

- Accession codes, unique identifiers, or web links for publicly available datasets
- A description of any restrictions on data availability
- For clinical datasets or third party data, please ensure that the statement adheres to our policy

Key sources such as genotyping, RT-qPCR primers and antibodies are provided in the method section. All relevant data supporting the key findings of this study are available within the article and its supplementary information files or from the corresponding author upon reasonable request. A reporting summary for this article is available as a supplementary information file. Source data are provided within this paper.

Research involving human participants, their data, or biological material

Policy information about studies with human participants or human data. See also policy information about sex, gender (identity/presentation), and sexual orientation and race, ethnicity and racism.

Reporting on sex and gender	The human kidney was removed in the setting of a tumor resection, the healthy (tumor free) part of kidney not needed for pathological diagnosis was used. Patients provided written consent for the use of samples for research. Because patients were anonymized, information on age and gender are not available.
Reporting on race, ethnicity, or other socially relevant groupings	The kidney samples are completely anonymized, no characteristics are available.
Population characteristics	Not available.
Recruitment	Not available.
Ethics oversight	Sample collection was not done by the investigators and all data are anonymized; therefore, they are exempt from IRB approval (§12 HambKHG).

Note that full information on the approval of the study protocol must also be provided in the manuscript.

Field-specific reporting

Please select the one below that is the best fit for your research. If you are not sure, read the appropriate sections before making your selection.

☒ Life sciences ☐ Behavioural & social sciences ☐ Ecological, evolutionary & environmental sciences

For a reference copy of the document with all sections, see nature.com/documents/nr-reporting-summary-flat.pdf

Life sciences study design

All studies must disclose on these points even when the disclosure is negative.

Sample size	Sample size per experiment was determined depending on<br>1) Experimental feasability<br>2) Mouse breeding- and genotype availability<br>In general, 2-3 independent experiments were pooled to reach statistical power in cases where experimental feasability did not allow experimentation of a sufficient n for statistical power.
Data exclusions	Mice were excluded depending on<br>1) the efficiency of transgene expression and<br>2) tubular contamination of glomerular preparations

Replication	All experiments were independently replicated over 2 times by different researchers and over the course of 6 years. The number of replications for each experiment is indicated in the figure legend
Randomization	Allocation to the groups occured based on the genotype
Blinding	Aquisition of histological measurements were performed in a blinded fashion. Investigators doing the data analysis were unaware of group allocations during data analysis.<br>The aquisition of animal data were performed in a blinded manner, revelation of genotypes and pharmacologic treatment occurred after data aquisition.

Reporting for specific materials, systems and methods

We require information from authors about some types of materials, experimental systems and methods used in many studies. Here, indicate whether each material, system or method listed is relevant to your study. If you are not sure if a list item applies to your research, read the appropriate section before selecting a response.

Materials & experimental systems

n/a	Involved in the study	
☐	☒	Antibodies
☐	☒	Eukaryotic cell lines
☒	☐	Palaeontology and archaeology
☐	☒	Animals and other organisms
☒	☐	Clinical data
☒	☐	Dual use research of concern
☒	☐	Plants

Methods

n/a	Involved in the study	
☒	☐	ChIP-seq
☐	☒	Flow cytometry
☒	☐	MRI-based neuroimaging

Antibodies

Antibodies used	Primary antibodies used for the study were: rabbit anti-ubiquitin (immunofluorescence microscopy (IF) 1:300, Novus, #NB300-129); guinea pig anti-nephrin (IF 1:200, Origene, #BP5030); rabbit anti-ß5c (IF 1:300, WB 1:5000, laboratory stock X. Wang, University of South Dakota, USA); rabbit anti-a2 (WB 1:1000, Cell Signaling, #2455); rabbit anti-a-actinin-4 (WB 1:1000, Immunoglobe, #0042-05); rabbit anti-p62 (WB 1:2000, Sigma-Aldrich, #P0067), guinea pig anti-p62 (IF 1:500, ProGene, #GP62-c); goat anti-Mrc2 (WB 1:1000, IF 1:200 R&D Systems, #AF4789); AF488 mouse anti H2Kd-(FACS-sort 1:250, BioLegend, Clone #SF1-1.1); BV421 rat anti-CD31 (FACS-sort 1:800, BD Horizon, #562939); AF647 rat anti-CD73 (FACS-sort 1:2000, BioLegend, #127208); AF700 rat anti-CD73 (FACS-sort 1:2000, BioLegend, #127230); PE hamster anti-podoplanin (FACS-sort 1:200, BioLegend, #127408); AF700 rat anti-CD45 (FACS-sort 1:100, BioLegend, #103128); APC-Cy7 rat anti-CD45 (FACS-sort 1:100, BioLegend, #103116); FITC mouse anti-pig CD31 (IF 1:30, Bio-Rad, #MCA1746F); goat anti-Biotin (WB 1:1000, ThermoFisher, #PA1-26792); AF546 rat anti-endomucin (IF 1:50, Santa Cruz, #sc-65495); rabbit anti-K48pUb (IF 1:300, WB 1:1000, Abcam, #ab140601); rabbit anti-LC3B (WB 1:5000, IF 1:50, Cell Signaling, #3868S) rabbit anti-Limp2 (WB 1:500, IF 1:1000, laboratory stock P. Saftig, CAU Kiel, Germany), rabbit anti-Lamp2 (WB 1:1000, IF 1:300, Sigma-Aldrich, #L0668), mouse anti-human LAMP1 (IF 1:200, DSHB, # H4A3); rabbit anti-p57 (IF 1:400, Santa Cruz, #sc-8298); rhodamine-wheat germ agglutinin (IF 1:400, WGA, Vector, #RL-1022); DyLight649-lycopersicon esculentum (tomato) lectin (i.v. injection of 10 ml of a 40 µg/ml solution, Vector, #DL-1178-1); DyLight488-Phalloidin (IF 1:200, Molecular Probes, #21833); AF488 donkey anti-rabbit IgG (IF 1:200, Cy2, WB 1:1000, Jackson ImmunoResearch, #711-545-152); Cy5 donkey anti-mouse IgG (IF 1:200, Cy5, Jackson ImmunoResearch, #715-175-151); guinea pig anti-synaptopodin (IF 1:400, Synaptic Systems, #163004); goat anti-collagen 4 (IF 1:400, Southern Biotechnologies, #1340-01); mouse anti-Rpt5 (WB 1:1000, Enzo, #BML-PW8770); rabbit anti-Lmp7 ((ß5i) WB 1:5000, laboratory stock E. Krüger, IF 1:300); mouse anti-ß-actin (WB 1:10000, Sigma-Aldrich, #A5441); goat anti-cathepsin D (WB 1:100, Santa Cruz, #sc-6486); rabbit anti-calreticulin (WB 1:500, Abcam, #ab92516). All secondary antibodies used were either biotinylated, HRP- or fluorescent dye-conjugated affinity purified donkey antibodies (Jackson ImmunoResearch).
Validation	We thoroughly describe the protocol for the usage of antibodies in our manuscript in the method section. All antibodies were validated as follows:<br>1) Beta5i antibodies were knockout validated by us.<br>2) All other antibodies were validated within the experiments by use of positive and negative controls by us.<br>3) All other antibodies used were either company or publication validated or validated by us using isotype controls.

Eukaryotic cell lines

Policy information about cell lines and Sex and Gender in Research

Cell line source(s)	Human immortalized podocytes (a kind gift of Moin Saleem, University of Bristol, Bristol, Great Britain; Saleem, M.A., et al. A conditionally immortalized human podocyte cell line demonstrating nephrin and podocin expression. J Am Soc Nephrol 13, 630-638 (2002).)<br>Primary podocytes were generated by standard procedures from murine glomerular outgrowths.

Authentication	Human and murine podocytes are authenticated based on their expression of podocyte proteins podocin and synaptopodin at the timing of experiments.
Mycoplasma contamination	Human podocytes were checked every 3 months for mycoplasma contamination. No contamination was present.
Commonly misidentified lines (See ICLAC register)	No commonly misidentified cell lines were used in the study.

Animals and other research organisms

Policy information about studies involving animals; ARRIVE guidelines recommended for reporting animal research, and Sex and Gender in Research

Laboratory animals	Mice used in the study were older than 8 weeks of age and were predominantly analyzed at 10 - 20 weeks of age. Mice were kept at ambient temperature (20-24 °C) and humidity (45 - 65 %), had free access to water and standard animal chow standard animal chow (Altromin 1328 P) and were synchronized to a 12h light : 12h dark cycle.<br><br>Male BALB/c mice were purchased from Charles River. For inhibitor studies mice received the proteasomal inhibitor epoxomicin (0.5 µg/g bodyweight) or the lysosomal inhibitor leupeptin A (40 µg/g bodyweight) by intra-peritoneal injection on 4 consecutive days, vehicle control mice received equal amounts (25%) DMSO in PBS. For quantification of glomerular immunoglobulin deposition, mice received 160 µl rbIgG (Bio & Sell) by intra-venous injection, 7 days post rbIgG injection mice received the aforementioned inhibitors on 4 consecutive days. Animal euthanasia was performed following subcutaneous buprenorphine (0,1 mg/kg KG) administration for analgesia 30 minutes prior to cervical neck dislocation under 3.5% isoflurane inhalation narcosis. All experimental procedures were performed according to the institutional guidelines.<br><br>Lmp7DEnC mice: Lmp7fl/fl mice were generated in C57BL/6J background by genOway (Lyon, France) by Prof. Dr. Frank Heppner, Charité Berlin, Germany. For the generation of an inducible endothelial cell-specific [illegible]5i-deficiency, Lmp7fl/fl mice were crossed to the Cdh5-Cre-ERT2 mouse line (Wang, Y., et al. Ephrin-B2 controls VEGF-induced angiogenesis and lymphangiogenesis. Nature 465, 483-486 (2010)). C57BL/6 Lmp7fl/fl cre- mice exposed to tamoxifen were used as control littermates (Ctrl). The naïve phenotype of mice was analyzed 5-50 weeks after the last tamoxifen injection.
Wild animals	Our study did not involve wild animals
Reporting on sex	Male mice were used for the inhibitor treatment studies.<br>Male and female mice were used for the Lmp7DEnC naive studies
Field-collected samples	Our study did not involve field-collected samples
Ethics oversight	The animal studies were conformed to the requirements of the German Animal Welfare Act and approvals were obtained from the State Authority of Hamburg (Behörde für Justiz und Verbraucherschutz, Amt für Verbraucherschutz, Lebensmittelsicherheit und Veterinärwesen), Germany.

Note that full information on the approval of the study protocol must also be provided in the manuscript.

Flow Cytometry

Plots

Confirm that:

- ☐ The axis labels state the marker and fluorochrome used (e.g. CD4-FITC).
- ☐ The axis scales are clearly visible. Include numbers along axes only for bottom left plot of group (a 'group' is an analysis of identical markers).
- ☐ All plots are contour plots with outliers or pseudocolor plots.
- ☐ A numerical value for number of cells or percentage (with statistics) is provided.

Methodology

Sample preparation	Kidney packages were taken from the mice. Glomeruli were isolated via Dynabead perfusion and magnetic enrichment. Single cell suspensions were obtained using a two-hour enzymatic digestion with Liberase and DNAse at 37°C. Over these two hours, glomeruli were repeatedly mechanically stressed to facilitate dissociation. Cells were stained for thirty minutes at 4°C, resuspended in PBS and strained through 40 µm cell strainers before sorting.
Instrument	Samples were analysed (and sorted, if applicable) at one of the following instruments: BD FACSAria Fusion, BD FACSAria IIIu, BD FACSymphony A3, BD LSR Fortessa.
Software	Flow cytometry data was collected using BD FACSDiva. Analysis was done using FlowJo. Statistical tests were performed in GraphPad Prism.
Cell population abundance	The endothelial cells analysed here make up the large majority of collected glomerular cells (>150000 endothelial cells

Cell population abundance	compared to <50000 podocytes and mesangial cells). The validity of the applied gating strategy was described in https://doi.org/10.1681/asn.2020091346. Endothelial cells analysed in this manuscript were repeatedly confirmed to carry the endothelial-specific loss of Lmp7.
Gating strategy	Cells were gated in an SSC-A vs FSC-A gate. Subsequently, dubletts were excluded using both an FSC-H vs FSC-W and SSC-H vs SSC-W gate. Dead cells were excluded with a live/dead stain (APC/Cyanine7 vs FSC-A plot). Now, mesangial cells (CD73-AF700 positive) were separated from Podocytes and Endothelial cells (CD31-BV421 positive). These CD73 positive mesangial cells were then freed from contaminating podocytes using a CD73-AF700 vs Podoplanin-PE plot. Podocytes and Endothelial cells were separated using a Podoplanin-PE vs CD31-BV421 gate. Cell populations could be clearly separated using the respective gates.

☐ Tick this box to confirm that a figure exemplifying the gating strategy is provided in the Supplementary Information.
